# Retinal microvascular and microstructural alterations in the diagnosis of dermatomyositis: a new approach

**DOI:** 10.3389/fmed.2023.1164351

**Published:** 2023-05-25

**Authors:** Bo-Zhi Huang, Qian Ling, San-Hua Xu, Jie Zou, Miao-Miao Zang, Xu-Lin Liao, Hong Wei, Ping Ying, Chong-Gang Pei, Yi Shao

**Affiliations:** ^1^Department of Ophthalmology, The First Affiliated Hospital of Nanchang University, Nanchang, Jiangxi, China; ^2^Department of Ophthalmology and Visual Sciences, The Chinese University of Hong Kong, Hong Kong, Hong Kong SAR, China

**Keywords:** interstitial lung disease, optical coherence tomography angiography, retinal thickness, vessel density, dermatomyositis (DM)

## Abstract

**Purpose:**

To study the relationship between fundus alterations, including retinal thickness and microvascular changes, and dermatomyositis (DM) using optical coherence tomography angiography (OCTA).

**Methods:**

A total of 16 patients with DM (32 eyes) and 16 healthy controls (HCs; 32 eyes) participated in this study. Based on the Early Treatment Diabetic Retinopathy Study subzones, OCTA fundus data were divided into different layers and regions for comparison.

**Results:**

The full retinal thickness (RT) in the inner nasal (IN), outer nasal (ON), inner inferior (II), and outer inferior (OI) regions of patients with DM was significantly lower than that of HCs (*P* < 0.001). The inner layer RT was also significantly lower in the IN, ON, II, and OI regions in patients with DM (*P* < 0.001). The outer layer RT was lower only in the II region in patients with DM compared to HCs (*P* < 0.001). The full RT of the II region was more sensitive to the pathological changes of disease since its ROC curve had an AUC of 0.9028, 95% CI: 0.8159–0.9898. Meanwhile, the superficial vessel density (SVD) of patients with DM was significantly lower in the IN, ON, II, and OI regions compared to HCs (*P* < 0.001). The AUC for region II was 0.9634 (95% CI: 0.9034–1.0), which indicated good diagnostic sensitivity.

**Conclusion:**

Optical coherence tomography angiography can be used to evaluate relevant ocular lesions and monitor disease progression in patients with DM and interstitial lung disease.

## Introduction

Dermatomyositis (DM) is one of the idiopathic inflammatory myopathies (IIM) and is uncommon in the general population. Epidemiological studies have shown that the incidence of IIM ranges from 2.47 to 7.8 per 100,000 person-years and the prevalence from 9.54 to 32.74 per 100,000 person-years (1–3). DM is the most common IIM (4), with an estimated incidence of 5–8.9/100,000 in adults (5). The sex ratio is approximately 1 (male): 2 (female) (4), and quite a few adult patients are diagnosed in their 40s−60s (5).

The clinical features are mainly Gottron's papules above bony prominences, progressive symmetrical proximal muscle weakness, and heliotrope rash on the eyelids (5). Discussing the diagnosis and classification of IIMs is difficult since they have different classification systems and diagnostic criteria at different times. The first diagnostic criteria for DM were proposed by Bohan and Peter in 1975 (6), and there have been many new classification systems proposed since then (7–11). In recent years, the first validated classification indicator has been proposed by the European League Against Rheumatism/American College of Rheumatology (EULAR/ACR) with a diagnostic sensitivity of 87% and a specificity of 82% (12). However, it is less sensitive to amyopathic DM (13). Additionally, the guide does not include any myositis-specific antibodies (MSAs) except anti-Jo-1 (14). Nonetheless, antibodies to melanoma differentiation-associated gene 5 (MDA5), an RNA unwinding enzyme involved in the viral immune response (15), are detectable in most cases of amyopathic DM and approximately 10–30% of patients with DM (4). Anti-MDA5 DM is commonly found in Asian populations, with some regional and ethnic differences (16, 17). Patients with this subtype of DM are predisposed to interstitial lung disease (ILD), which can progress rapidly under some circumstances (15, 18, 19). The associated 6-month mortality rate is almost 59%; therefore, early recognition can make a significant difference in disease treatment (20).

Idiopathic inflammatory myopathies do not involve only the muscles, but often also the skin, joints, and eyes (including involvement of adjacent structures, such as damage to the anterior and posterior segments) (21). The earliest report of DM-related retinopathy was the study by Bruce (22), and subsequent reports of ocular symptoms associated with DM have been published (23–26). However, only a few cases of Purtscher-like retinopathy (PLR) have been reported in recent years (27, 28). Although retinal involvement is not uncommon in DM, case reports and studies of ocular symptoms in DM are scarce, and PLR has hardly been reported, specifically in anti-MDA5 DM. Anti-MDA5 DM has some distinctive cutaneous manifestations, which studies suggest are the result of cutaneous vascular lesions (29, 30). These manifestations have been seen as characteristic markers of anti-MDA5 DM; however, eye lesions have not been adequately studied and reported. Cutaneous injury and retinopathy in anti-MDA5 DM share a common mechanism; the degree of visual loss from retinopathy depends on the blood vessels involved and the degree of ischemia (27, 31).

Eye lesions are often part of various immune-related systemic diseases and connective tissue disorders. Complicated eye disease in DM can be an indicator of disease progression but is often overlooked. Optical coherence tomography angiography (OCTA) is non-invasive and provides detailed microvasculature information (32). In some ways, it can be said that OCTA is the best means of non-invasively examining microvascular changes in the eye.

There are no studies that have systematically evaluated OCTA findings in patients with DM; furthermore, even the identified ocular changes have been poorly reported. In our study, we aimed to investigate ocular changes in patients with DM assessed by OCTA, such as retinal thickness (RT) and vessel density (VD).

## Methods

### Subjects

This clinically controlled study was conducted from 1 January 2021 to 31 December 2021, at the Department of Ophthalmology and Rheumatology of the First Affiliated Hospital of Nanchang University. A total of 32 participants were recruited; 16 patients with DM were recruited from the rheumatology outpatient clinic and 16 healthy controls matched for age and sex were recruited from the Ocular Disease Clinical Research Center. All subjects were clinically examined by an ophthalmologist from the research center, and OCTA imaging was performed to assess the eyes for abnormalities.

### Inclusion and exclusion criteria

All patients were randomly selected from existing outpatient cases, and the inclusion criteria were as follows: (1) DM at the active phase diagnosed according to EULAR/ACR criteria (12) and (2) combined ILD with clinical symptoms and signs, such as dyspnea and cough, and confirmed by pulmonary function tests and computerized tomography (CT) imaging.

Exclusion criteria included the following: (1) other autoimmune diseases, including systemic sclerosis; (2) systemic diseases, including neurological diseases which would affect the eye and optic nerve; (3) retinal pathology, such as glaucoma and arteriovenous disease; (4) history of ocular trauma/surgery; (5) other diseases affecting fundus imaging; and (6) pregnant and breastfeeding women.

### Ethical statement

This study was conducted following the tenets of the Declaration of Helsinki and was approved by the Ethics Committee of the First Affiliated Hospital of Nanchang University. Every participant fully understood the study methods before participating, was aware of the possible risks, and finally, agreed to participate in the study. All participants provided written informed consent.

### Clinical examinations

Patients underwent the following clinical and ophthalmic examinations: (1) clinical examination according to EULAR/ACR criteria, including muscle biopsy; (2) immunological tests, such as complement and antibody numbers, tests for 16 MSAs; (3) pulmonary function tests and CT lung examination to confirm the state of ILD; (4) routine blood tests to assess inflammatory status; (5) Hospital Anxiety and Depression Scale (HADS) to evaluate mental status; (6) basic eye examination including Snellen visual acuity (VA), spherical equivalent, intraocular pressure (IOP) measured with Goldmann applanation tonometer, and dry eye evaluation; and (7) OCTA.

### Ocular surface evaluation

Tear breakup time (BUT) was evaluated after the uniform application of sodium fluorescein to the ocular surface. The time to initial tear film breakup after a blink was observed under the cobalt blue light. The tear breakup time was considered positive when it was < 10 s.

A combination of corneal fluorescein staining and conjunctival lysine green staining was used to evaluate the ocular staining score (OSS). An OSS score ≥ 3 was considered positive.

To perform the Schirmer's test (SIT) without anesthesia, one end of 5 × 35 mm filter paper was folded at a right angle, sterilized, and placed in the conjunctival sac. An SIT < 5 mm after 5 min was considered positive.

For the tear meniscus height (TMH), we used infrared light to focus and asked the patient to blink. Then, the TMH was measured and recorded with the Keratograph 5M after white light exposure.

### Optical coherence tomography angiography

The RTVue Avanti XR system (Optovue, Fremont, CA) for OCTA imaging shows both retinal sections and the microvasculature. Scanning parameters were set in accordance with those reported by Ye (33). The specific settings used in this study were as follows: scan speed 70,000 A-scans per second; central wavelength 840 nm; bandwidth 45 nm; horizontal resolution 22 μm; and axial resolution 5 μm. We performed five consecutive angiograms using the 6 × 6 mm scan pattern, with B-scans along the X-axis and 216 raster positions along the Y-axis, with a focus on the fovea. The results of 1080 B-scans (216y × 5) were obtained at 270 frames per second. The whole scan duration was 3.9 s, and a three-dimensional 3 × 3 mm OCTA image of each eye was obtained.

Based on the Early Treatment Diabetic Retinopathy Study (ETDRS) delineation of retinal subzones (three concentric circles with radii of 0.5, 1.5, and 3 mm), we divided the retina into nine regions and analyzed their thickness. The nine retinal regions analyzed were the inner nasal (IN), outer nasal (ON), inner inferior (II), outer inferior (OI), inner temporal (IT), outer temporal (OT), inner superior (IS), outer superior (OS), and foveal center (C). The inner RT was the thickness from the internal limiting membrane (ILM) to the inner plexiform layer, and the full RT was the thickness from the ILM to the retinal pigment epithelium (RPE). The calculated difference between the two numbers (full RT and inner RT) was the outer RT. We calculated the vessel density from the center of the macula to the edge of the 3 mm × 3 mm luminance gradient image via model construction (34), measuring the macular retinal thickness and the superficial vessel density (SVD). The data were obtained from the right eye of all participants. Therefore, data from the left eye were flipped. The two sets of data were averaged and processed for analysis together.

### Statistical analysis

Data were analyzed using GraphPad Prism version 9 (La Jolla, California, USA) and reported as mean ± standard deviation (SD). Independent-samples *t*-test, chi-square test, and Fisher's exact test were used to analyze the data between the DM and HCs groups. Snellen visual acuity was converted to logMAR values for analysis. Differences in RT and SVD between the DM and HCs groups were analyzed using generalized estimating equations, and data were adjusted to account for intrasubject interocular correlations using known confounding variables. We performed univariate and multivariate regressions to analyze the relationship between the RT thickness and systemic and ocular variables. Differences in SVD and RT between HCs and patients with DM were analyzed by receiver operating characteristic (ROC) curves. The relationship between RT and SVD was analyzed by linear regression. We considered a *P*-value < 0.05 to be statistically significant.

## Results

### Subjects

A total of 32 participants were enrolled in this study, 16 patients with DM (32 eyes) and 16 healthy controls (32 eyes). There was no statistically significant difference in age (*P* = 0.745) or sex composition (*P* = 1.000) between the DM and HCs groups. There was also no significant difference in blood pressure between the two groups. However, the HADS scores of the DM group were significantly higher than those of HCs (DM: 7.50 ± 2.73 vs. HCs: 2.81 ± 1.11; *P* < 0.001) (Table 1).

**Table 1:** Basic information on subjects.

	**DM**	**HCs**	***t***	***P*-value**
Age (y)	48.75 ± 6.93	49.50 ± 5.96	0.329	0.745^a^
Sex (female:male)	7:01	7:01	-	1.000^b^
Duration of DM (m)	24.50 ± 39.25	N/A	-	-
ESR (mm)	33.75 ± 25.83	N/A	-	-
CRP (10mg/L)	6.24 ± 3.71	N/A	-	-
CK-MB	32.32 ± 21.16	N/A	-	-
MDA-5, *n* (%)	14 (87.5)	N/A	-	-
FVC (L), **%**	2.935 ± 0.26, 72.42 ± 16.35	N/A	-	-
RV (L), %	1.83 ± 0.42, 118.94 ± 26.48	N/A	-	-
TLC (L), %	5.16 ± 0.99, 83.93 ± 9.82	N/A	-	-
DLCO (mol/min/KPa), %	7.74 ± 0.39, 63.29 ± 8.04	N/A	-	-
SBP (mmHg)	122.19 ± 13.19 124.44 ± 5.74	0.626	0.536^a^
DBP (mmHg)	84.50 ± 6.46	82.94 ± 6.30	0.693	0.494^a^
HADS	7.50 ± 2.73 2.81 ± 1.11	6.358	< 0.001^a^

^a^Independent-samples t-test; ^b^chi-square test. Bolded % is the ratio of measured to predicted values. CK-MB, creatine kinase MB isoenzymes; CRP, C-reactive protein; DBP, diastolic blood pressure; DLCO, diffusion capacity for carbon monoxide of lung; DM, dermatomyositis; ESR, erythrocyte sedimentation rate; FVC, forced vital capacity; HADS, Hospital Anxiety and Depression Scale; HCs, healthy controls; MDA5, melanoma differentiation-associated protein 5; N/A, not applicable; SBP, systolic blood pressure; TLC, total lung capacity.

Compared to HCs, patients with DM had lower VA (0.46 ± 0.13 vs. 0.90 ± 0.09; *P* < 0.001), lower SIT scores (6.25 ± 0.73 vs. 12.94 ± 0.85 mm; *P* < 0.001), lower TMH (0.15 ± 0.02 vs. 0.58 ± 0.10 mm, *P* < 0.001), shorter BUT (5.00 ± 0.98 vs. 13.91 ± 1.58 s; *P* < 0.001), and higher OSS scores (1.78 ± 0.60 vs. 0; *P* < 0.001); meanwhile, the IOP was similar (15.98 ± 1.56 vs. 15.23 ± 1.49 mmHg; *P* = 0.173) (Table 2). However, multivariate regression analysis showed that there was no clear correlation between RT and the basic parameters of patients with DM mentioned above (Table 3).

**Table 2:** Ocular conditions of subjects.

	**DM**	**HCs**	***P*-value^a^**
IOP (mmHg)	15.98 ± 1.56	15.23 ± 1.49	0.17
VA (logMAR)	0.46 ± 0.13	0.90 ± 0.09	< 0.001
tBUT(s)	5.00 ± 0.98	13.91 ± 1.58	< 0.001
OSS	1.78 ± 0.0.60	0	< 0.001
SIT (mm)	6.25 ± 0.73	12.94 ± 0.85	< 0.001
TMH (mm)	0.15 ± 0.02	0.58 ± 0.10	< 0.001

^a^Independent-samples t-test. DM, dermatomyositis; HCs, healthy controls; IOP, intraocular pressure; OSS, ocular staining score; SIT, Schirmer test; tBUT, tear breakup time; TMH, tear meniscus height; VA, visual acuity.

**Table 3:** Multivariate regression analysis between macular retinal thickness and demographic and ocular parameters in patients with DM.

	**Multivariate regression analysis Regression coefficient (β ±SE)**	***P*-value^a^**
Age (month)	0.851 ± 0.374	0.023
VA (logMAR)	19.533 ± 18.097	0.280
IOP (mmHg)	−0.379 ± 1.192	0.751
SBP (mmHg)	0.176 ± 0.115	0.125
DBP (mmHg)	0.195 ± 0.422	0.643

^**a**^Generalized estimating equation (right eyes involved). DBP, diastolic blood pressure; IOP, intraocular pressure; SBP, systolic blood pressure; VA, visual acuity.

### Macular retinal thickness

**Figure 1:**
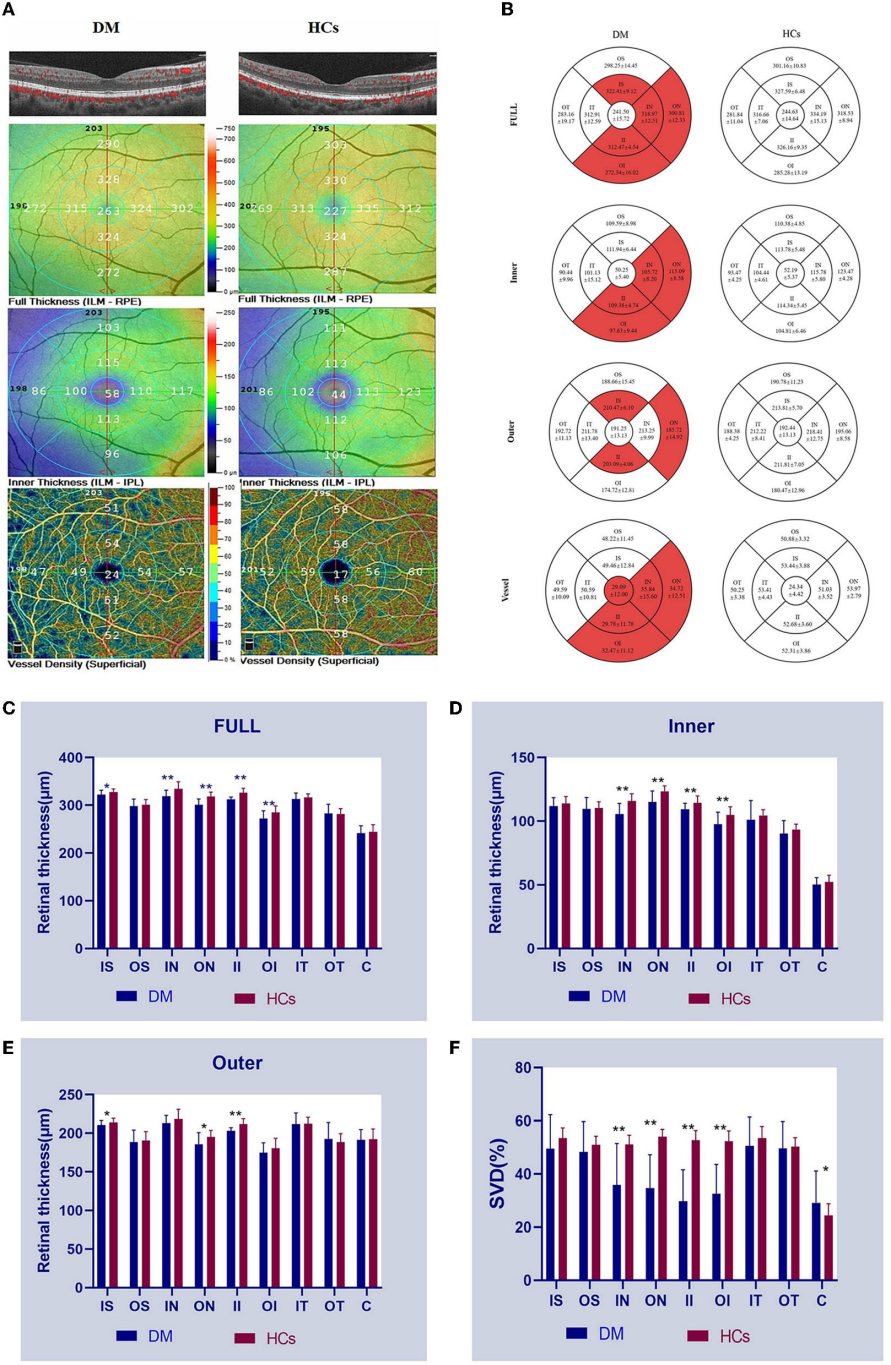
OCTA images and RT and SVD analysis of the anti-MDA5 DM and HCs group. **(A)** View of RT and SVD in anti-MDA5 DM and HCs by OCTA. The inner RT, full RT, and SVD were classified based on ETDRS criteria. **(B–F)** The results of inner RT, outer RT, full RT, and SVD in the anti-MDA5 DM and HCs group are compared. anti-MDA5 DM, anti-melanoma differentiation-associated protein 5 dermatomyositis; C, center; ETDRS, early treatment diabetic retinopathy study; HCs, healthy controls; II, inner inferior; IN, inner nasal; IT, inner temporal; IS, inner superior; OCTA, optical coherence tomography angiography; OI, outer inferior; ON, outer nasal; OS, outer superior; OT, outer temporal; RT, retinal thickness; SVD, superficial vessel density. ^*^p < 0.1; ^**^p < 0.001.

The comparison of RT between the DM and HCs groups is shown *in* Figures 1A, B and Table 4. The full RT in the IN, ON, II, and OI regions of patients with DM was significantly lower than those of HCs (*P* < 0.001) (Figure 1C). No significant differences (*P* > 0.05) existed in any of the other regions (IT, OT, IS, OS, and foveal center). The inner RT was also significantly lower in the IN, ON, II, and OI regions in patients with DM compared to HCs (*P* < 0.001) (Figure 1D). Other regions of the inner ring were similarly not significantly different from the HCs group (*P* > 0.05). In the outer layer, only the RT of region II in patients with DM was significantly lower than HCs (*P* < 0.001), while all other regions were similar to HCs (*P* > 0.05) (Figure 1E).

**Table 4:** Comparison of macular retinal thickness at different locations between patients with DM and HCs.

**Location**	**DM (n=16,32 eyes)**	**HCs (n=16,32 eyes)**	***P*-value^a^**
**Macular full retinal thickness (**μ**m, mean** ±**SD)**			
IS	322.41 ± 9.12	327.59 ± 6.48	**0.011**
OS	298.25 ± 14.45	301.16 ± 10.83	0.366
IN	318.97 ± 12.51	334.19 ± 15.13	**< 0.001**
ON	300.81 ± 12.33	318.53 ± 8.94	**< 0.001**
II	312.47 ± 4.54	326.16 ± 9.35	**< 0. 001**
OI	272.34 ± 16.02	285.28 ± 13.19	**< 0.001**
IT	312.91 ± 12.59	316.66 ± 7.06	0.147
OT	283.16 ± 19.17	281.84 ± 11.04	0.738
C	241.50 ± 15.72	244.63 ± 14.64	0.414
**Macular inner retinal thickness (**μ**m, mean** ±**SD)**			
IS	111.94 ± 6.44	113.78 ± 5.48	0.222
OS	109.59 ± 8.98	110.38 ± 4.85	0.667
IN	105.72 ± 8.20	115.78 ± 5.80	**< 0.001**
ON	115.09 ± 8.58	123.47 ± 4.28	**< 0.001**
II	109.38 ± 4.74	114.34 ± 5.45	**< 0.001**
OI	97.63 ± 9.44	104.81 ± 6.46	**< 0.001**
IT	101.13 ± 15.12	104.44 ± 4.61	0.240
OT	90.44 ± 9.96	93.47 ± 4.25	0.118
C	50.25 ± 5.40	52.19 ± 5.37	0.155
**Macular outer retinal thickness (**μ**m, mean** ±**SD)**			
IS	210.47 ± 6.10	213.81 ± 5.70	**0.027**
OS	188.66 ± 15.45	190.78 ± 11.23	0.531
IN	213.25 ± 9.99	218.41 ± 12.75	0.077
ON	185.72 ± 14.92	195.06 ± 8.58	**0.003**
II	203.09 ± 4.06	211.81 ± 7.05	**< 0.001**
OI	174.72 ± 12.81	180.47 ± 12.96	0.079
IT	211.78 ± 13.40	212.22 ± 8.41	0.884
OT	192.72 ± 11.13	188.38 ± 4.25	0.308
C	191.25 ± 13.13	192.44 ± 13.13	0.721

^a^Independent-samples t-test. Bold indicates *P* < 0.05. C, center; DM, dermatomyositis; HCs, healthy controls; II, inner inferior; IN, inner nasal; IS, inner superior; IT, inner temporal; OI, outer inferior; ON, outer nasal; OS, outer superior; OT, outer temporal.

### Superficial macular retinal vascular density

The SVD of the different retinal regions can be seen in Figures 1A, B, and Table 5. The SVD of patients with DM can be seen to be significantly lower in the IN, ON, II, and OI regions compared to HCs (*P* < 0.001) (Figure 1F).

**Table 5:** Comparison of superficial vessel density at different locations between patients with DM and HCs.

**Location(mean ±SD)**	**DM (n=16,32 eyes)**	**HCs (n=16,32 eyes)**	***P*-value^a^**
IS	49.46 ± 12.84	53.44 ± 3.88	0.099
OS	48.22 ± 11.45	50.88 ± 3.32	0.212
IN	35.84 ± 15.60	51.03 ± 3.52	**< 0.001**
ON	34.72 ± 12.51	53.97 ± 2.79	**< 0.001**
II	29.78 ± 11.78	52.68 ± 3.60	**< 0.001**
OI	32.47 ± 11.12	52.31 ± 3.86	**< 0.001**
IT	50.59 ± 10.81	53.41 ± 4.43	0.178
OT	49.59 ± 10.09	50.25 ± 3.38	0.728
C	29.09 ± 12.00	24.34 ± 4.42	**0.039**

^a^Independent-samples t-test. Bold indicates *P* < 0.05. C, center; DM, dermatomyositis; HCs, healthy controls; II, inner inferior; IN, inner nasal; IS, inner superior; IT, inner temporal; OI, outer inferior; ON, outer nasal; OS, outer superior; OT, outer temporal.

### ROC curve analysis of RT and SVD

**Figure 2:**
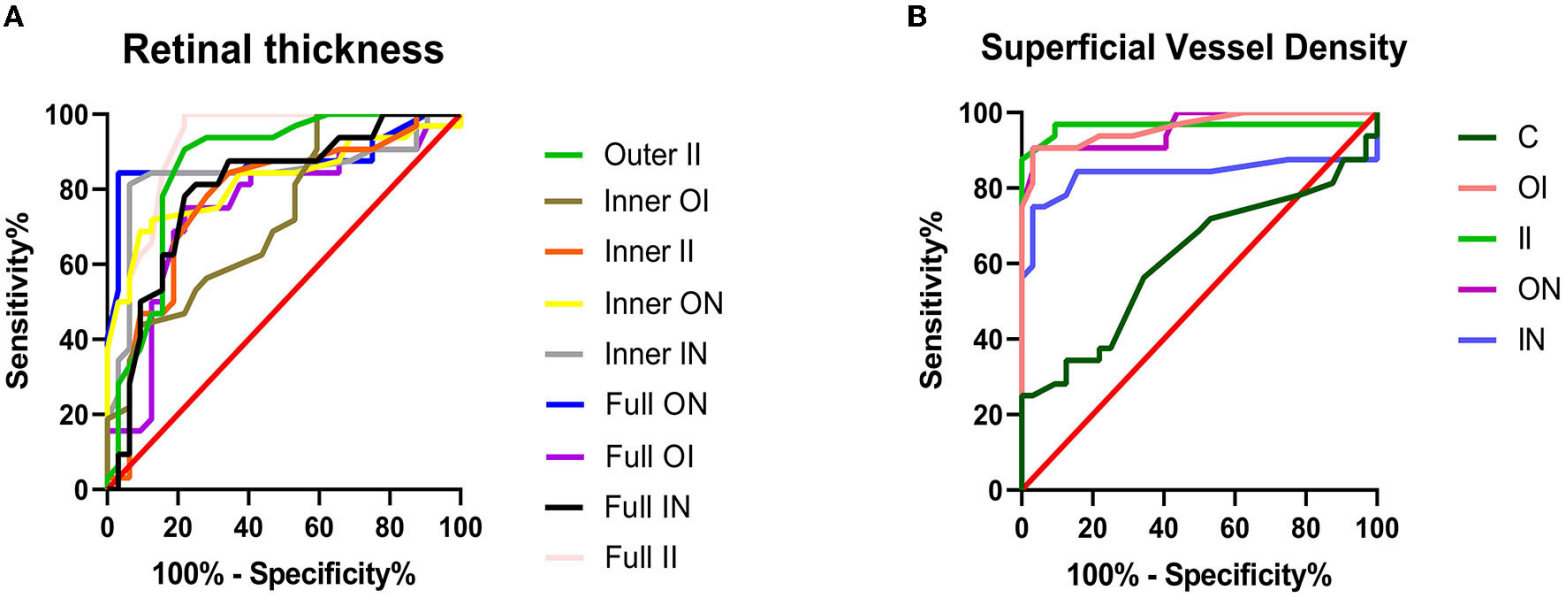
ROC curve analysis of retinal thickness and superficial vessel density. **(A)** The area under the curve (AUCs) for RT ROC curves were as follows: full II = 0.9028 (95% CI: 0.8159–0.9898); full IN = 0.8018 (95% CI: 0.6891–0.9144); full OI = 0.7490 (95% CI: 0.6227–0.8753); full ON = 0.8765 (95% CI: 0.7792–0.9737); inner II = 0.7769 (95% CI: 0.6577–0.8960); inner IN = 0.8408 (95% CI: 0.7306–0.9510); inner OI = 0.7310 (95% CI: 0.6094–0.8526); inner ON = 0.8228 (95% CI: 0.7159–0.9296); and outer II = 0.8628 (95% CI: 0.7669–0.9587). **(B)** The AUCs for SVD ROC curves were as follows: C = 0.6133 (95% CI: 0.4722–0.7544); II = 0.9634 (95% CI: 0.9034–1.0); IN = 0.8379 (95% CI: 0.7193–0.9565); OI = 0.9619 (95% CI: 0.9180–1.0); and ON = 0.9575 (95% CI: 0.9108–1.0). C, center; CI, confidence interval; II, inner inferior; IN, inner nasal; IS, inner superior; OI, outer inferior; ON, outer nasal; ROC, receiver operating characteristic; RT, retinal thickness; SVD, superficial vessel density.

The sensitivity and specificity of parts of regions were evaluated, as it is known that the RT or SVD of those regions is lower compared to HCs (Figures 2A, B). The area under the curve (AUC) for the Full II RT ROC curve was 0.9028 (95% CI: 0.8159–0.9898) and AUC for the II SVD ROC curve was 0.9634 (95% CI: 0.9034–1.0). It could be concluded that the II region indicated the highest diagnostic sensitivity among all regions.

### Relationship between RT, SVD, duration, and HADS

**Figure 3:**
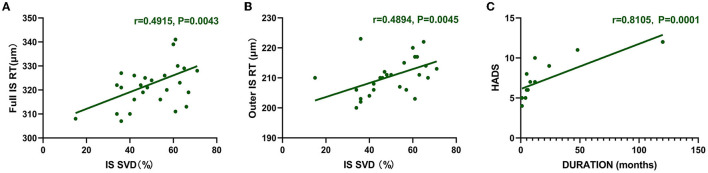
Correlation between RT and SVD. HADS was correlated with duration. **(A, B)** The vertical coordinate is the value of RT (μm), and the horizontal coordinate is the value of SVD (%). In the DM group, full RT and outer RT were positively correlated with SVD at IS region (full RT: *r* = 0.4915, *P* = 0.0043; outer RT: *r* = 0.4894, *P* = 0.0045). **(C)** Positive correlation between duration and HADS (*r* = 0.8105, *P* = 0.0001). The vertical coordinate is the value of HADS, and the horizontal coordinate is the duration. DM, dermatomyositis; HADS, Hospital Anxiety and Depression Scale; IS, inner superior; RT, retinal thickness; SVD, superficial vessel density.

In DM patients, full RT and outer RT of the IS region are correlated with SVD (full RT: *r* = 0.4915, *P* = 0.0043; outer RT: *r* = 0.4894, *P* = 0.0045) (Figures 3A, B). The longer the course of anti-MDA5 DM, the higher the HADS index (*r* = 0.8105, *P* = 0.0001) (Figure 3C).

## Discussion

As a type of connective tissue disease (CTD), DM may be associated with a series of systemic manifestations, including typical muscle involvement and ocular disorders. Characteristic ocular involvement includes anterior inflammation (heliotropic eyelid rash) and posterior inflammation (retinal hemorrhages and cotton wool spots). DM may also present ocular symptoms, such as redness and vision loss, similar to other CTDs (35). Clinically, the relationship between CTDs and ocular alterations cannot be ignored; therefore, ideally, ocular examinations should be included in the management of systemic diseases. In most cases, fluorescein angiography has been considered a standard in ocular disease diagnosis; however, its invasive nature limits its use. Specific populations, especially patients who suffer from nephropathy and serious hypertension, could not undergo this test at frequent follow-up intervals. OCTA provides a more detailed display of retinal and choroidal blood flow than fluorescence (36). In some cases, the fundus alterations recorded by OCTA can indicate the early phase of the disease and be used to monitor disease progression. It has been reported that lower RT and SVD occurred in patients with Sjögren's syndrome, systemic lupus erythematosus, and Behcet's disease (34, 37, 38). Our results show that patients with DM presented similar results. Both the full RT and inner RT were lower in the IN, ON, II, and OI regions of patients with DM (*P* < 0.001). The SVD of patients with DM was significantly lower in the IN, ON, II, and OI regions compared to HCs (*P* < 0.001). Full RT and outer RT were positively correlated with SVD at the IS region. The SVD of the II region was more sensitive to the pathological changes of disease since its ROC curve had an AUC of 0.9634 (95% CI: 0.9034–1.0). The AUC for the full RT of region II also indicated a higher diagnostic sensitivity (0.9028, 95% CI: 0.8159–0.9898). Recently, a study that aimed to investigate fundus microvasculature of juvenile dermatomyositis revealed that there was no significant difference in SVD between the HC and DM groups (39). But the vessel density in deep vessel density (DVD) of DM was significantly lower than that of HCs (39). Based on the study of juvenile DM and our investigation, it can be concluded that the fundus microvasculature had a decreasing tendency in the progression of the disease. The discrepancy in results may be due to the distinction of manifestations among different populations or the different stages of the disease. Several studies of CTDs have found that the involvement of DVD occurs earlier than that of SVD (34, 40, 41). In the future, longitudinal studies defining the stages of the disease may be more convincing. The contrast is also more meaningful.

Anyhow, changes in macular thickness and SVD could be considered important indicators for evaluating DM progression according to our research. The development of DM could induce retinal artery or vein occlusion and nervous system lesions, which can present as macular edema, subretinal fluid, high reflection in the retinal neuroepithelium layer, and thin retinal nerve fiber layer (42). OCTA technology can be used to evaluate vessel density and even the severity of occlusion. The significance of improving OCTA in early diagnosis and follow-up was confirmed in monitoring ischemic involvement leading to irreversible visual loss. Microstructural changes are mainly concentrated in the temporal and inferior macular retina, which should be paid more attention in clinical practice. In future studies, the generality and individuality of retinal parameter changes in different CTDs and details of the underlying mechanisms can be studied.

Griger et al. (43) showed that the corneas of patients with DM and polymyositis were thinner and had lower volumes, as observed by Pentacam. In our study, patients with DM had lower VA, SIT scores, and TMH and shorter BUT, which supports the known high prevalence of dry eye disease (DED) in DM. Some researchers have insisted that there is an association between corneal abnormalities and autoantibodies, as corneal tissue is avascular (44). The corneal epithelium contains Langerhans cells, lymphopoietin, and immune factors, which develop an inflammatory response; thus, cellular immunity mediated by T cells and other immunomodulatory factors contribute to the creation of keratopathy. Furthermore, DED is not only associated with the cornea but also with the conjunctiva. Chen et al. (45) used functional slit-lamp biomicroscopy to compare the conjunctival blood flow of patients with DED and HCs and concluded that patients with DED have higher vessel density and larger vessel diameter (45). It could be speculated that the same findings would be present in patients with DM, and OCTA technology may also be used to detect conjunctival microvascular changes when the focus is set on the anterior segment.

All the subjects had interstitial lung disease, and 87.5% of them were anti-MDA5 DM. ILD is considered an earlier manifestation of DM, and 78% of patients with ILD show specific symptoms, such as ground-glass lung consolidations (46). At the cellular immunity level, pulmonary pathology in DM is associated with an increase in CD8+ and CD68+ T cells (47). Up to 90% of the population who are anti-Jo-1 antibody positive suffers from ILD (48). For patients with DM and ILD, skin lesions, lung disease, and changes in ocular microvessels are caused by IFN-1 (29). IFN-1 could cause damage to the endothelium and induce local ischemia, especially in patients with anti-MDA5 DM (49). Some studies have proposed that there is a specific pathway in anti-MDA5 DM to trigger the increase of IFN-1. The severity of the disease is associated with antibody titer (50), as anti-MDA5 antibodies are associated with a rapidly progressive disease phenotype. It has been known that anti-MDA5 DM is susceptible to systemic multi-organ involvement. Hence, fundamental research on the relationship between IFN-1 and fundus microvascular alterations or lung injury in DM would make sense. In terms of treatment, anti-MDA5 DM, unlike other types of DM, is sensitive to therapy that combines tacrolimus with cyclophosphamide. This should be referred to in future studies of pathological mechanisms.

In addition to physical abnormalities, patients with CTDs also present psychological problems. Some patients with rheumatoid arthritis or multiple sclerosis present with mental illness. Psychological disorders interact with the autoimmune disease and affect the speed of disease development and treatment response. It has been reported that anxiety and depression are common in patients with juvenile myositis (51). However, there have been few investigations on the mental health of patients with DM. In this study, patients with DM had significantly higher HADS scores than HCs (DM: 7.50 ± 2.73 vs. HCs: 2.81 ± 1.11; *P* < 0.001). In addition, HADS increased with the prolongation of disease duration (*r* = 0.8105, *P* = 0.0001). This corroborates the psychological problems mentioned above. It would be interesting to identify the relevant mechanism in a follow-up study.

There are a few limitations in our study. First, the results from a small sample size such as ours need to be verified in future studies. Second, other factors that could affect RT and VD should be considered. For example, patients under different therapeutic schedules were not distinguished; therefore, the influence of drugs could not be excluded. Finally, in this study, all participants with DM had ILD and the partial pressure of oxygen in patients with ILD is lower, which could influence ocular blood vessels. Thus, the lung disorder itself could lead to retinal microvascular disease (52). In the future, studies involving patients with DM without ILD should be performed.

## Conclusion

Our OCTA investigation revealed that the RT and SVD of specific subregions in the macular retina of patients with DM were lower than those of HCs. Abnormal VA, SIT scores, TMH, and BUT explain the high prevalence of DED in patients with DM. This study provides a new direction for the treatment of DM.

## Data availability statement

The raw data supporting the conclusions of this article will be made available by the authors, without undue reservation.

## Ethics statement

The studies involving human participants were reviewed and approved by the Ethics Committee of the First Affiliated Hospital of Nanchang University. The patients/participants provided their written informed consent to participate in this study.

## Author contributions

Material preparation, data collection, and analysis were performed by B-ZH, S-HX, X-LL, and M-MZ. YS and C-GP are the guarantors of the integrity of the entire study. The first draft of the manuscript was written by B-ZH and JZ. The statistical analysis was performed by QL. Clinical data were collected by JZ, B-ZH, S-HX, QL, and PY. Literature research was performed by X-LL, HW, M-MZ, and PY. All authors contributed to the conception, design of the study, commented on previous versions of the manuscript, read, and approved the final manuscript.
